# Dermoscopy in Monitoring Treatment Response in Scabies: A Scoping Review

**DOI:** 10.3390/jcm15187085

**Published:** 2026-09-12

**Authors:** Mateusz Krzysztof Mateuszczyk, Magdalena Łyko, Joanna Maj

**Affiliations:** University Centre of General Dermatology and Oncodermatology, Faculty of Medicine, Wroclaw Medical University, Borowska 213, 50-556 Wrocław, Poland

**Keywords:** scabies, dermoscopy, treatment monitoring, ultraviolet-induced fluorescence dermoscopy, scoping review

## Abstract

**Background**: Scabies is a World Health Organization–designated neglected tropical disease of rising incidence with increasingly reported treatment failure, making objective verification of cure important. Dermoscopy is well established for diagnosis, but its role in assessing treatment response has never been mapped. **Objectives**: To map how dermoscopy assesses treatment response in scabies—which markers, timepoints and cure definitions are applied, and how it relates to clinical and microscopic standards. **Methods**: A scoping review following PRISMA-ScR searched PubMed/MEDLINE, Web of Science, EBSCO and Scopus (2016–2026, English) using a two-concept strategy (scabies × dermoscopy); eligibility required baseline dermoscopy plus at least one further, separately reported dermoscopic assessment during or after therapy. Two reviewers screened independently, supplemented by citation searching. **Results**: Ten studies were included. The same mite structure appeared under at least four names; assessment timepoints (day 2 to beyond day 28) and cure definitions were inconsistent. Dermoscopy was the genuine object of investigation in few studies; only one used ultraviolet-induced fluorescence to track response. **Conclusions**: Despite the disease’s recognised importance, dermoscopic monitoring of scabies remains a declared rather than a designed research aim. No standardised marker, timepoint or cure definition exists; consensus standards analogous to the 2020 IACS diagnostic criteria are needed.

## 1. Introduction

Scabies, caused by *Sarcoptes scabiei* var. *hominis*, affects an estimated 200 million people worldwide and was designated a neglected tropical disease by the World Health Organization (WHO) in 2017 [1]. Incidence has risen in high-income European countries over the past decade, and reports of failure of first-line topical permethrin have accumulated [2,3], with debate over whether these reflect true pharmacological resistance, pseudo-resistance or operational factors. Whatever the mechanism, the practical consequence is the same: after treatment, clinicians increasingly need an objective way to determine whether infestation has been eradicated.

Dermoscopy has become the standard non-invasive method of visualising the mite in vivo. The 2020 International Alliance for the Control of Scabies (IACS) consensus criteria [1] place direct visualisation at the highest level of diagnostic certainty (confirmed scabies, level A), but they distinguish sharply between modalities. Light microscopy of skin samples (subcategory A1) and non-invasive high-powered imaging devices offering magnification of at least ×70, such as videodermoscopy or reflectance confocal microscopy (A2), may confirm the diagnosis through the mite, its eggs or its faecal pellets. Dermoscopy (A3), by contrast, requires definitive visualisation of the mite itself; where only a burrow is seen, the criteria permit a diagnosis of clinical scabies (B1) but not of confirmed scabies.

Beyond diagnostic certainty, dermoscopy is attractive as a point-of-care tool: it is rapid and non-invasive, requires neither skin sampling nor a laboratory microscope, and can be performed at the bedside with an inexpensive handheld device. This is especially valuable where conventional light microscopy is unavailable or impractical—rural and remote clinics, refugee and migrant camps, and institutional outbreaks, settings in which much of the global scabies burden falls—and in young children, in whom dermoscopy is far easier to perform than obtaining skin scrapings for microscopy. Its principal limitations are operator-dependence and a learning curve for recognizing the mite and its structures, and the fact that its confirmatory value depends on visualising the mite itself—under the IACS criteria, a burrow alone supports only a clinical, not a confirmed, diagnosis [1]. These strengths and constraints, well characterized for diagnosis, frame the question this review addresses: whether dermoscopy can serve equally well for confirming cure.

The diagnostic role of dermoscopy is therefore well documented; its role after treatment is far less clear. The contrast is striking for a disease of the global public-health importance underscored by the WHO designation; no agreed objective test confirms that treatment has worked. The most comprehensive review of dermoscopic monitoring of therapeutic response in general dermatology [4] devotes a section to scabies but anchors it on two primary studies from 2001 and 2004 [5,6], and a 2026 topical review [7] still rests on the same pre-2016 anchors—a field that has grown in volume without methodological consolidation. We therefore undertook a scoping review to map how dermoscopy is used to assess treatment response in scabies. Four mapping sub-questions addressed (i) which dermoscopic markers distinguish active from cured infestation; (ii) at which timepoints assessment is performed and how dermoscopic cure is defined; (iii) in which treatment regimens and study designs dermoscopy is applied; and (iv) how dermoscopic assessment agrees with clinical and microscopic confirmation of cure.

## 2. Materials and Methods

### 2.1. Protocol and Registration

The review was conducted and reported in accordance with the Preferred Reporting Items for Systematic Reviews and Meta-Analyses extension for Scoping Reviews (PRISMA-ScR) [8]. A scoping design was chosen because the research question is a mapping question—twhich markers, at which timepoints, in which contexts—rather than a question about the effect of a defined intervention, and because the anticipated heterogeneity of designs and outcome definitions precluded quantitative synthesis. Because the aim was to map the evidence rather than to estimate the effect of a defined intervention, the review question was framed with the Population–Concept–Context (PCC) framework rather than a PICO question, which does not apply to a scoping review. The protocol was registered on OSF Registries (Open-Ended Registration; registered 25 June 2026; DOI 10.17605/OSF.IO/2GH63). Registration was concurrent; the preliminary counts in the registered protocol were superseded by the definitive search of 30 July 2026 (Section 3.1). PROSPERO does not accept scoping reviews.

### 2.2. Eligibility Criteria

Eligibility was framed using the Population–Concept–Context (PCC) framework (Table 1). The operational inclusion criterion was that a study must report a baseline dermoscopic assessment together with at least one further dermoscopic assessment at a timepoint after the start or completion of therapy, serving to establish activity or resolution of the infestation. A study qualified whether or not dermoscopy formally defined cure, provided it reported a dermoscopic result distinct from the other methods used (microscopy or clinical assessment) at a timepoint during or after treatment; studies in which dermoscopy was folded into a composite endpoint together with those methods, with no separately reported dermoscopic result, were not eligible. Purely diagnostic studies, studies monitoring response with another modality without a dermoscopic component, studies of post-scabetic pruritus without assessment of mite activity, non-English-language studies, retracted studies and secondary literature were excluded. Secondary sources were used only as a starting point for citation searching and in the discussion.

### 2.3. Information Sources and Search Strategy

Four databases were searched and limited to the 2016–2026 window and to English-language records: PubMed/MEDLINE, Web of Science Core Collection, EBSCO Academic Search Ultimate and Scopus. All four databases were searched on 30 July 2026 and de-duplicated together as a single pool; the counts reported here reflect that search date.

A deliberate two-concept search strategy (scabies × dermoscopy) was used, without a third concept of “monitoring” or “treatment response”. Including such a concept as a search filter would have risked missing studies that perform dermoscopic assessment after treatment but do not use that monitoring vocabulary in the title, abstract or keywords. The monitoring requirement was therefore not applied as a title/abstract filter but verified at the full-text assessment stage: at title/abstract screening, records were excluded only when clearly ineligible (not scabies, no dermoscopy, or no treatment component), while any record whose treatment-monitoring role could not be excluded from the title or abstract was carried forward to full text. Full-text assessment was therefore applied only to records retrieved by the two-concept (scabies × dermoscopy) search that had survived title/abstract screening; it was not an independent full-text search of the entire scabies literature. This preserved maximal sensitivity, at the cost of a larger number of records to screen than a three-concept search (adding a “monitoring”/“treatment response” filter) would have returned. The full verbatim search strings for all four databases, with per-database record counts, are provided in Table S1 (Supplementary Materials).

### 2.4. Selection of Sources of Evidence

Titles and abstracts were screened independently by two reviewers to remove clearly ineligible records; the full eligibility criteria, including the treatment-monitoring requirement, were then applied at full-text assessment, also in duplicate, following a calibration exercise on a sample of records. Inter-rater agreement at the full-text level was quantified using Cohen’s κ, interpreted per Landis and Koch [9]; the record that could not be retrieved was excluded from this calculation. Disagreements were to be resolved by consensus, with a third reviewer acting as arbiter where consensus could not be reached.

The database search was supplemented by citation searching in two directions: backward citation, comprising the bibliography of the most relevant review of dermoscopic treatment monitoring and the reference lists of all included studies, and forward citation, comprising studies citing the included studies, with particular attention to recent publications on scabies dermoscopy with a declared response-assessment component and to the UVFD literature.

### 2.5. Data Charting and Synthesis

Data were charted into a single extraction sheet by one reviewer and verified by a second. Charted variables were study design, dermoscopy modality (device type, magnification, use of UV), population, treatment regimen, assessment timepoints, the role played by dermoscopy in the study, the unit and object of measurement, and the definition of cure. A second sheet charted, marker by marker, the dermoscopic appearance at baseline and its change at the reported post-treatment timepoint(s)—during treatment and/or at cure, depending on the study. Results are presented as a narrative synthesis structured by the four sub-questions, together with tabulated study characteristics. Formal appraisal of methodological quality or risk of bias was not performed, in line with PRISMA-ScR guidance for scoping reviews.

## 3. Results

### 3.1. Selection of Sources of Evidence

**Figure 1:**
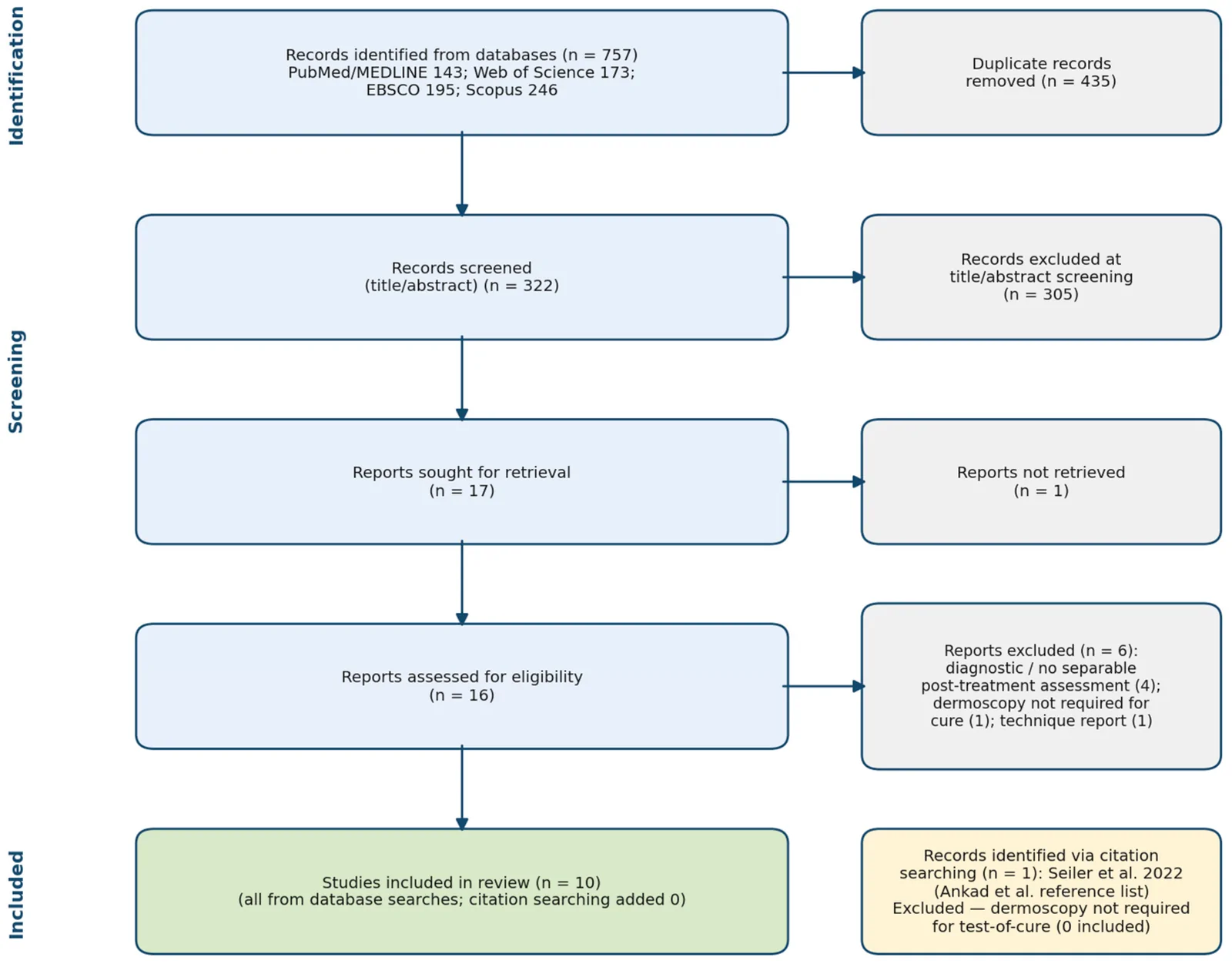
PRISMA-ScR flow diagram of the study-selection process.

The four database searches, run on 30 July 2026, yielded 757 records (PubMed/MEDLINE 143, Web of Science 173, EBSCO 195, Scopus 246), of which 435 were duplicates, leaving 322 unique records for title/abstract screening. Of these, 305 were excluded at title/abstract screening and 17 reports were sought for full-text assessment; one could not be obtained in full text and is reported separately as “not retrieved”. Of the 16 database reports assessed for eligibility, 6 were excluded: 4 purely diagnostic or providing no separable post-treatment dermoscopic assessment, one interventional case series with no dermoscopic result reported separately from microscopy, and one technique report without a post-treatment dermoscopic timepoint [10]. Ten studies were included, all from the database searches; backward citation searching identified one further in-window report, but it did not meet the inclusion criteria, so no study was added, giving 10 included studies. Inter-rater agreement at the full-text level was Cohen’s κ = 1.00 (100% agreement across 16 scored records), with no disagreements requiring arbitration. Counts at each stage are given in Table 2; a graphical PRISMA-ScR flow diagram is shown in Figure 1 [11].

### 3.2. Characteristics of Included Studies

The 10 included studies comprised randomised controlled trials, prospective and retrospective observational cohorts, a diagnostic-accuracy study, an interventional case series, a technique report and a case report. Across these designs, the scabicides evaluated were permethrin, benzyl benzoate, oral ivermectin, lindane, sulfur and spinosad [2,3,12,13,14,15,16,17,18,19]. Five studies performed at least one dermoscopic assessment at an intermediate timepoint during treatment (serial monitoring) and five assessed only baseline and at-cure status. Full characteristics, including dermoscopy modality, population, treatment regimen, assessment timepoints, the role of dermoscopy and the definition of cure, are given in Table 3; the marker-by-marker dermoscopic trajectory is given in Table 4.

### 3.3. Dermoscopic Markers (Sub-Question 1)

Across the ten studies the same mite structure is reported under at least four names—the “jet with contrail” sign [12,14], the “delta-wing” or “triangular delta” sign [15,17], the “kite sign” [2,3,16] and the “brownish triangular mite head” [19]—so that nomenclature, rather than the sign itself, is the main source of heterogeneity. The burrow is the next most frequent marker. Eggs and scybala are seldom scored separately and are most often subsumed within a composite cure criterion. Morphological mite viability is tracked in only one study [13], which describes progressive degradation of the dark delta wing into a translucent “dry hydrangea” appearance as evidence of mite death. Ultraviolet-induced fluorescence is used to track response in only one study [17], in which it is the disappearance after treatment of the greenish mite fluorescence and of the bluish-white burrow fluorescence—in parallel with loss of the polarised delta sign—that is reported as an effective marker of response, although the fluorescence signal was not assessed independently of the polarised examination.

### 3.4. Timepoints and Definition of Cure (Sub-Question 2)

Assessment timepoints range from days 2 and 4 after treatment, through weekly serial visits, to a single day-28 endpoint or a week-3 visit. Definitions of dermoscopic cure are correspondingly inconsistent: some studies accept dermoscopic negativity alone, others use dermoscopy interchangeably with microscopy within a composite “microscopic cure”, and one large cohort [18] treats dermoscopy as confirmatory to a clinical endpoint, though it still reported a separable dermoscopic result at test-of-cure (residual dermoscopic positivity in a subset despite clinical cure). One randomised trial [3] applies an explicitly asymmetric rule, in which dermoscopic persistence of mites counts automatically as treatment failure whereas dermoscopic absence alone is not accepted as cure. Two distinct conceptual levels of assessment coexist without being reconciled: a binary presence/absence criterion consistent with the IACS framework, and a morphological assessment of mite viability.

### 3.5. Treatment Regimens and Study Designs (Sub-Question 3)

Dermoscopy is the genuine object of investigation in only a minority of studies. In the randomised trials it functions as a binary endpoint attached to a scabicide-efficacy question. Intermediate-timepoint (serial) monitoring appears in studies ranging from single case reports to larger observational cohorts, whereas the largest randomised trials assess only baseline and final status.

### 3.6. Agreement with Clinical and Microscopic Reference Standards (Sub-Question 4)

Most studies treat dermoscopy as interchangeable with microscopy and therefore report no concordance data. The single explicit discordance comes from a prospective multicentre cohort [18] in which 3 of 47 patients judged clinically cured were dermoscopy-positive at day 28. This is precisely the kind of finding a monitoring tool is meant to surface—a possible residual infestation not detected by clinical assessment—yet no included study was designed or powered to confirm it.

### 3.7. Studies Excluded at Full Text, Not Retrieved, and Citation Searching

Six reports were excluded at full text (Table 5): four were purely diagnostic or provided no separable post-treatment dermoscopic assessment [20,21,22,23], one interventional case series that reported no dermoscopic result distinct from microscopy (dermoscopy interchangeable with microscopy, not performed in all patients, the marker unspecified) [24], and one recent technique report that used ultraviolet-induced fluorescence to assess mite viability but reported no separable dermoscopic assessment at a defined post-treatment timepoint [10]. One report remains not retrieved [25] and may be eligible.

Backward citation searching identified one additional in-window report—Seiler et al. 2022 [26], from the reference list of Ankad et al. [17]—which did not meet the inclusion criteria: the trial reported no separable dermoscopic result. Complete cure required both a clinical and a microscopic cure; within the microscopic-cure component the absence of mites/eggs/scybala could be shown by microscopy or dermoscopy interchangeably (with a negative dermoscopy result required for burrows), but no separable dermoscopic appearance, morphology or trajectory is described. Dermoscopy thus functions only as a binary element of a composite cure endpoint, not as a monitoring assessment establishing activity or resolution at any timepoint during or after treatment. No eligible study was therefore added through citation searching. Notably, the scabies section of the most comprehensive review of dermoscopic treatment monitoring rests on two primary studies published in 2001 and 2004 [5,6], both outside the search window. Forward citation searching identified no new eligible study; the studies retrieved were diagnostic comparisons, case reports with clinical-only follow-up, or trials without a dermoscopic timepoint, all of which were also captured by the database search. Verdicts for all reports assessed through backward and forward citation searching are provided in Table S2.

## 4. Discussion

This scoping review maps a literature in which dermoscopic monitoring of scabies treatment response is a declared aim rather than a designed one: studies announcing “treatment monitoring” were found at full text to perform diagnostics. The topic’s core also predates the search window—the most comprehensive review of dermoscopic response monitoring [4] still anchors its scabies section on primary studies from 2001 and 2004 [5,6], and the more recent 2026 review [7] rests on the same anchors—indicating a field that has grown in volume but not in methodological consolidation.

Monitoring as a primary research aim is almost unstudied. Of the 10 included studies, dermoscopy is the genuine object of investigation in only a few; elsewhere it is a binary endpoint of scabicide trials or cohorts. Only five of the ten studies performed dermoscopic assessment at an intermediate timepoint during treatment, and these range from single case reports to larger observational cohorts, whereas the largest randomised trials assess only baseline and final status. Prospective studies designed for the purpose of monitoring, rather than borrowing dermoscopy as a convenient endpoint, remain absent.

No standardised marker, timepoint or level of assessment exists. Assessment points diverge from day 2 to day 28 and beyond; the same dermoscopic sign carries multiple names; and definitions of dermoscopic cure are not comparable across studies. Most importantly, two non-integrated paradigms of assessment coexist—a binary presence/absence criterion and a morphological assessment of mite viability—and the same dermoscopic event can therefore support opposite operational rules, as in the asymmetric failure criterion applied by one trial. This maps onto the IACS diagnostic hierarchy—even though those criteria were designed for diagnosis, not for monitoring cure: a mite still visualised after treatment meets the level A (confirmed) threshold and so objectively confirms that the mite is still present, whereas a negative dermoscopic examination would, by the same logic, return the assessment to clinical (level B) status and cannot, on its own, confirm cure. Crucially, this is a diagnostic mapping: it certifies presence, not viability, and therefore does not by itself prove that infestation is still active—the distinction developed below. One of the largest spinosad trials—excluded here because it reported no dermoscopic result distinct from the other methods (dermoscopy folded into a composite binary endpoint)—nonetheless names four dermoscopic markers within that cure definition and describes none of them, scoring them only as a composite binary criterion [26]. The absence of an agreed marker, timepoint and level of assessment is the natural target for a consensus process analogous to the role of the 2020 IACS criteria in diagnosis [1].

Fluorescence-based monitoring is essentially untested. UVFD has grown rapidly in the scabies literature since 2023 [27,28], but almost exclusively in a diagnostic context. In this review only one study used UVFD to document treatment response [17], reporting loss of the green mite fluorescence in parallel with disappearance of the polarised delta sign. The dynamics of the fluorescence signal during and after therapy—whether it disappears as rapidly as, or more rapidly than, the polarised signs—remain practically unexamined and represent a well-defined direction for further work.

Where dermoscopy has disagreed with the clinical reference, the discordance may be clinically relevant: the 3 of 47 dermoscopy-positive “clinical cures” reported in one cohort [18] could reflect residual infestation not detected clinically—potentially the added value a monitoring tool should provide, yet one that no included study was powered to test. Clinically, this suggests that dermoscopic confirmation of cure may identify patients at risk of apparent relapse or continued transmission; methodologically, it identifies concordance between dermoscopic, microscopic and clinical assessment as a priority endpoint for future studies.

The pediatric dimension deserves particular emphasis. Only one included study concerned an infant—serial subungual dermoscopic monitoring in a child with crusted scabies [14]—yet scabies in infants is a frequent and clinically distinctive entity, prone to diagnostic delay and misdiagnosis, in which dermoscopy is already the practical diagnostic workhorse: in a recent Spanish single-centre series of 51 infants, 22 of the 23 confirmed diagnoses were established by dermoscopy rather than microscopy [29]. Extending this same, well-tolerated bedside tool from diagnosis to the monitoring of cure is therefore a natural and clinically valuable step, but one that needs explicit validation in young children, in whom skin sampling for microscopy is particularly difficult and a rapid, non-invasive confirmation of cure would be of greatest value.

Underlying all of this is a more fundamental limitation: dermoscopy demonstrates the mite’s presence rather than its viability. Loss of green fluorescence has been proposed as a sign of a dead mite [10], but beyond that proposal it remains unvalidated as a criterion of cure; the ‘dry hydrangea’ appearance of a degrading mite [13] seems a more convincing morphological marker of death, yet it is discernible only at the high magnification of videodermoscopy—impractical for the whole-body examination that cure assessment demands, since a single viable mite anywhere constitutes treatment failure. Establishing cure therefore calls for prospectively validated markers designed for that purpose—potentially features beyond visualising the mite itself, such as the loss of eggs or of burrow-associated signs—assessed across the whole body in a way feasible at the point of care, a task better suited to handheld than to video dermoscopy.

The clinical implication is therefore not a ready-made monitoring protocol but a defined research agenda. A usable standard will have to specify which dermoscopic marker denotes residual activity, at which timepoint it is assessed, how final clearance is confirmed, and at what level of certainty—clinical or imaging-confirmed, in the sense of the IACS hierarchy—cure is declared; the present evidence, spanning assessment points from day 2 to beyond day 28 with inconsistently named markers and a single study using UVFD to track response, cannot yet fix any of these.

### Strengths and Limitations

Strengths of this review include a deliberately sensitive two-concept search across four databases, duplicate screening with high inter-rater agreement, and supplementary citation searching in both directions, which surfaced no eligible study beyond the database corpus, supporting saturation.

Several limitations apply. Inclusion was restricted to English-language publications, which may exclude relevant non-English reports on scabies treatment monitoring. The corpus is heterogeneous in design, and in most studies dermoscopy functions only as a binary endpoint rather than the object of investigation. Formal risk-of-bias appraisal was not performed, as is standard for scoping reviews, so the mapping presented here describes the distribution of the evidence and not its quality. Heterogeneity of timepoints and cure definitions precluded any quantitative synthesis. One report could not be retrieved in full text and remains to be assessed. The 2016 lower bound of the search window was set deliberately rather than arbitrarily: it captures the contemporary literature that followed the consolidation of standardised dermoscopic diagnosis and the work of the IACS, so that the mapped evidence reflects current devices, terminology and diagnostic standards.

## 5. Conclusions

Across the 2016–2026 literature, dermoscopy in scabies is well documented as a diagnostic tool but remains an evidence deficit as a means of monitoring treatment response—a gap out of step with the disease’s WHO-recognised public-health importance and its rising incidence and treatment failure. Three gaps define the field: response monitoring is rarely a stated research aim; there is no agreed marker, timepoint or level of assessment for dermoscopic cure; and ultraviolet-induced fluorescence dermoscopy is described almost exclusively in a diagnostic context. These findings set clear directions for prospective studies purpose-designed to monitor dermoscopic treatment response, and for a consensus on assessment standards—ideally pursued through a structured Delphi process involving the IACS, the European Society for Dermatological Research (ESDR) and patient organizations—analogous to the 2020 IACS criteria for diagnosis.

## Figures and Tables

**Table 1:** Population–Concept–Context (PCC) framework and eligibility criteria.

Element	Definition
Population	Patients with confirmed scabies (clinical/dermoscopic/microscopic) undergoing scabicide treatment; any age and type.
Concept	Dermoscopy (polarised, ultraviolet-induced fluorescence dermoscopy (UVFD), videodermoscopy, ultra-high resolution) applied to assess treatment response during or after therapy.
Context	Any setting (outpatient, inpatient, institutional outbreaks); no geographic restrictions.

**Table 2:** Study selection: PRISMA-ScR counts at each stage.

Stage	Count
IDENTIFICATION	
Records from PubMed/MEDLINE	143
Records from Web of Science Core Collection	173
Records from EBSCO (Academic Search Ultimate)	195
Records from Scopus	246
Total identified	757
DE-DUPLICATION	
Duplicates removed	435
Records after de-duplication (title/abstract screening)	322
Excluded at title/abstract screening	305
FULL-TEXT ASSESSMENT (database records)	
Sought for full-text assessment	17
Not retrieved (Di Bartolomeo)	1
Excluded at full text (diagnostic/no separable post-treatment dermoscopy 4; dermoscopy not required for cure 1; technique report [Pietkiewicz 2026] 1)	6
Included via database searches	10
CITATION SEARCHING	
Included via backward citation searching	0
Total studies included	10

**Table 3:** Characteristics of the included studies, dermoscopy modality, role of dermoscopy and what was measured.

Study	Study Type	Dermoscopy (Type/Magnification/UV)	Patients (n; Notable Features)	Treatment + Regimen	Follow-Up Timepoints	Role of DS	Dermoscopic Findings Measured	Cure Definition
Li and Chen 2020 [12]	Diagnostic accuracy study	Dermat Image System (Dermat, Beijing); not specified whether handheld or video; 20× (screening)–50× (confirmation); use of UV not specified	N = 144	Sulfur ointment; concentration and regimen not specified	Days 2 and 4 after treatment	Diagnosis (final diagnosis required confirmation by light microscopy) + at test-of-cure timepoint	Absence/presence of jet with contrail and scybala	Not defined
Ma et al. 2026 [19]	Retrospective cohort with prospective dermoscopic monitoring sub-study	Dermoscope; handheld/video not specified; 20× magnification; UV not used	N = 100 (monitoring sub-study; dermoscopy-positive patients)	10% sulfur ointment (5% for children <3 yr)	Serial: ~10, 20, 30 and >30 days	Tool for the treatment endpoint (serial dermoscopic test-of-cure)	Presence/absence of burrows and the triangular mite head (per patient)	Dermoscopic negativity (no burrows/no triangular mite head); persistence → re-treatment
Mang et al. 2020 [13]	Technique description	Videodermoscopy; 30×–120×; use of UV not specified	Illustrative case material—no cohort	Scabicide unspecified	Every 24–48 h; ~7 days	Assessing mite viability	Morphology of mite body (intestine, legs, hairs, spines, bristles), burrow, eggs, scybala	Not applied
Jiang et al. 2022 [14]	Case report	Dermoscopy; ×60; no UV	N = 1, infant (6-month-old), crusted scabies with subungual involvement	Lindane 1% (2 days) → permethrin 5% (7 days, then 2× /week for 1 week)	Days 4, 7 and 11	Diagnosis (together with microscope) + at test-of-cure timepoint	Absence/presence of jet-with-contrail sign	Dermoscopy based: disappearance of mites (marker unspecified)
Yurekli et al. 2026 [15]	Prospective observational	Not specified; post-treatment dermoscopic figure at day 28	N = 76; only high-load phenotype (at least 50 dermoscopically mite-confirmed burrows per patient; non-crusted scabies)	Oral ivermectin 200 µg/kg (day 1 and 8) + topical permethrin 5% (day 1 and 8); ± cycle on day 14	day 14 and 28	Diagnosis + at test-of-cure timepoint	Mite load; absence/presence of delta-wing sign	Dermoscopic clearance: no mites/eggs and no new burrows.
Meyersburg et al. 2022 [2]	Randomised controlled trial	Not specified	N = 55	Topical permethrin 5% (2× within 1-week interval vs. additional local daily application)	Follow-up: week 3–4; total follow-up 6 weeks (assessments within this window not detailed)	Diagnosis + at test-of-cure timepoint	Mite density; absence/presence of mites (particular marker unspecified)	Dermoscopy based: not specified
Meyersburg et al. 2023 [16]	Randomised controlled trial	Not specified	N = 224	Topical benzyl benzoate 10/25% (3 days) vs. oral ivermectin 200 µg/kg 2× a week apart	Single follow-up: week 3–4	Diagnosis + at test-of-cure timepoint	Absence/presence of the kite sign	Not specified
Meyersburg et al. 2024 [3]	Randomised controlled trial	Not specified	N = 106	Topical permethrin 5% vs. topical benzyl benzoate 25%, daily ×3 days	Single: week 3 (+1 week if persistent eczema/itch)	Diagnosis + at test-of-cure timepoint	Mite density; absence/presence of kite sign, burrow	No mites in dermoscopy: marker unspecified + no eczema + no itch
Ankad et al. 2026 [17]	Interventional case series	Handheld dermoscopy (DermLite DL5); 10×; polarised + UVFD (UV mode)	N = 40	Spinosad 0.9% lotion once	Single follow-up at week 3	Diagnosis + at test-of-cure timepoint	Absence/presence of triangular delta sign, curvilinear tracks, and fluorescence	Not specified
Caumes et al. 2022 (EPIGALE) [18]	Prospective observational (multicentre)	Not specified	N = 130	Benzyl benzoate 10% emulsion; 2 applications 8 days apart	day 28 (clinical endpoint, dermoscopy) + telephone day 84	Diagnosis (one of the tools, next to clinical judgment and parasitological exam) + at test-of-cure timepoint	Absence/presence of a dermoscopic mite sign (not specified) at day 28	Pruritus reduction > 75% at day 28 vs. day 0 or VAS < 20 at day 28 + clinical assessment; cure did not necessarily require dermoscopy

**Table 4:** Dermoscopic trajectory of individual markers over time in the included studies.

Study	Active Infestation (Baseline Appearance)	Post-Treatment Change (Marker Change, with Timepoint)
Li and Chen 2020 [12]	Mite: jet with contrail sign Burrow: jet with contrail sign Eggs: not reported Scybala: used as indirect evidence of active disease UVFD: not reported	Mite: fewer affected sites at follow-up—assessment was location-based; the dermoscopic criterion for a “negative” site was not specified. In residual sites: less clearly visible, but the specific dermoscopic marker was not defined. Burrow: damaged/disrupted in persisting sites. Eggs: not reported Scybala: not reported UVFD: not reported
Ma et al. 2026 [19]	Mite: brownish triangular mite head present Burrow: sinuous burrows present Eggs: not reported Scybala: not reported UVFD: not reported	Mite: serial dermoscopic clearance is the negativity criterion (absence of burrows and triangular mite head); cure 13% by day 10. Cumulative negativisation 28%/32%/36% at the ~20/30/>30-day visits; 64% lost to follow-up. Burrow: absent at the test-of-cure timepoint in cured patients; persistence → second course. Eggs: not reported Scybala: not reported UVFD: not reported
Mang et al. 2020 [13]	Mite: movement of surface structures, head, legs or intestinal peristalsis; panoramic images every 1–2 days showing location changing Burrow: presence reported Eggs: presence reported Scybala: presence reported UVFD: not reported	Mite: viability tracked morphologically—alive = movement/peristalsis (see baseline appearance); death = degradation of the delta wing: “dry hydrangea” appearance; absence of movement/peristalsis (lack of detectable movement does not surely confirm death, since live mites do not always display those signs). Burrow: not reported Eggs: not reported Scybala: not reported UVFD: not reported
Jiang et al. 2022 [14]	Mite: presence of jet-with-contrail sign Burrow: presence of jet-with-contrail sign Eggs: not reported Scybala: not reported UVFD: not reported	Mite: absence of jet-with-contrail sign Burrow: absence of jet-with-contrail sign Eggs: not reported Scybala: not reported UVFD: not reported
Yurekli et al. 2026 [15]	Mite: present delta-wing sign Burrow: present delta-wing sign Eggs: not reported Scybala: not reported UVFD: not reported	Mite: persistence of the delta sign at day 14 triggered an additional treatment cycle (day 14 was a management visit, not an analytic endpoint); at day 28 resolution definition includes obligatory disappearance of the delta sign Burrow: resolution definition includes as obligatory empty, desquamating, “disrupted” roof at day 28 Eggs: based on dermoscopy clearance definition absent at day 28 Scybala: based on dermoscopy clearance definition absent at day 28 UVFD: same definition as not-UV mode
Meyersburg et al. 2022 [2]	Mite: present kite sign Burrow: present kite sign Eggs: not reported Scybala: not reported UVFD: not reported	Mite: kite sign less visible (“indistinct and pale”) after treatment; the authors attributed this to impaired mite mobility and explicitly stated that mite survival was not dramatically reduced (i.e., not attributed to mite death); significance for treatment response not established; mite density decreased from baseline to follow-up; not linked by the authors to response/non-response Burrow: disappearance Eggs: not reported Scybala: not reported UVFD: not reported
Meyersburg et al. 2023 [16]	Mite: present kite sign Burrow: present kite sign Eggs: not reported Scybala: not reported UVFD: not reported	Mite: marker unspecified, at week 3 dermoscopic persistence of mites was treated automatically as treatment failure Burrow: not reported Eggs: not reported Scybala: not reported UVFD: not reported
Meyersburg et al. 2024 [3]	Mite: present kite sign Burrow: present kite sign Eggs: not reported Scybala: not reported UVFD: not reported	Mite: marker unspecified; at week 3 absence of mites alone was not accepted as a cure (clinical criteria also had to be met), whereas dermoscopic persistence of mites alone was treated automatically as treatment failure Burrow: not reported Eggs: not reported Scybala: not reported UVFD: not reported
Ankad et al. 2026 [17]	Mite: present triangular delta sign (polarised) Burrow: present curvilinear white tracks (polarised) Eggs: not reported Scybala: not reported UVFD: green fluorescence of the mite; blue-white fluorescence of burrows	Mite: good response connected to disappearance of the delta sign Burrow: good response connected to disappearance of curvilinear tracks Eggs: not reported Scybala: not reported UVFD: good response connected to disappearance of green mite fluorescence and blue-white burrow fluorescence
Caumes et al. 2022 (EPIGALE) [18]	Mite: specific sign not reported Burrow: not reported Eggs: not reported Scybala: not reported UVFD: not reported	Mite: specific sign not reported Burrow: not reported Eggs: not reported Scybala: not reported UVFD: not reported

**Table 5:** Reports excluded at the full-text assessment stage, with reasons.

Study	Reason for Exclusion
Qaseem et al. 2024 [20]	Cross-sectional, purely diagnostic study (60 patients, Duhok). Despite a “treatment response” title, there is no dermoscopic assessment at a post-treatment timepoint; the declared R^2^ = 0.643 is not supported by the methods or the results.
Zaim et al. 2025 [21]	Diagnostic case report (annular scabies). The day-10 follow-up was purely clinical (pigmentation), with no repeat dermoscopy assessing mite activity—monitoring only declarative.
Boralevi et al. 2026 [22]	Efficacy RCT (oral ivermectin vs. permethrin 5%, classic scabies). Dermoscopy used for baseline diagnostic confirmation only; the authors explicitly performed no post-treatment dermoscopic assessment. No dermoscopic timepoint after the start of therapy—inclusion criterion not met.
Bernigaud et al. 2026 (GALE CRUSTED) [23]	Randomised trial in severe (profuse/crusted) scabies comparing higher- versus standard-dose oral ivermectin (400 vs. 200 µg/kg on days 0, 7 and 14), both combined with 5% permethrin cream. Cure was defined as absence of mites and mite-related products confirmed by “parasitologic or dermoscopic assessment” on days 18/21—dermoscopy fully interchangeable with parasitology, so cure could be established without any dermoscopic assessment. No dermoscopy-specific marker, baseline appearance or post-treatment trajectory is reported; the single mention of the “delta-wing jet” pattern is descriptive and cites prior work. The operational criterion—a separable baseline and post-treatment dermoscopic assessment—is therefore not met.
Komoda et al. 2021 [24]	Interventional case series (whole-body-bathing ivermectin; N = 7 elderly patients). Dermoscopy was used interchangeably with microscopy and was not performed in all patients; cure (“no mites” on microscopic or dermoscopic examination on two consecutive weekly tests, plus absence of new scabies lesions) could be established without dermoscopy. The marker was unspecified and no dermoscopic result was reported separately from microscopy at any timepoint during or after treatment—the operational criterion is not met.
Pietkiewicz et al. 2026 [10]	Technique report (JAAD “Technology Pearls”) demonstrating, on illustrative images, that magnified UVFD distinguishes viable (green fluorescent) from non-viable (“ghost”) mites. Fails the Concept criterion: it demonstrates the imaging technique rather than assessing treatment response and reports no separable baseline-to-post-treatment dermoscopic assessment tracked in a treated patient at a defined timepoint.

## Data Availability

All data charted in this review are contained within the article and its Supplementary Material. The review protocol is registered on the Open Science Framework (https://doi.org/10.17605/OSF.IO/2GH63).

## Supplementary Materials

The following supporting information can be downloaded at: https://www.mdpi.com/article/10.3390/jcm15187085/s1. Table S1: full electronic search strategy (as conducted) for all four databases; Table S2: Backward- and forward-citation searching (snowballing): Studies assessed and verdicts, including two reports assessed only at that stage [30,31]; PRISMA-ScR checklist.
