# Supplementary material for: Dermoscopy in Monitoring Treatment Response in Scabies: A Scoping Review
Source: J Clin Med. 2026 Sep 12;15(18):7085. doi: 10.3390/jcm15187085 (PMC13607758; doi:10.3390/jcm15187085)
Supplement: Supplementary file 1 [file jcm-15-07085-s001.zip › PRISMA-ScR Checklist.pdf]

PRISMA-ScR Checklist

Dermoscopy in Monitoring Treatment Response in Scabies: A Scoping Review

Section	Item	PRISMA-ScR Checklist Item	Reported on Page # / Section
**TITLE**			
**Title**	1	Identify the report as a scoping review.	Title page, p. 1
**ABSTRACT**			
**Structured summary**	2	Provide a structured summary that includes (as applicable): background, objectives, eligibility criteria, sources of evidence, charting methods, results, and conclusions that relate to the review questions and objectives.	Abstract, p. 1
**INTRODUCTION**			
**Rationale**	3	Describe the rationale for the review in the context of what is already known. Explain why the review questions/objectives lend themselves to a scoping review approach.	1. Introduction, p. 1
**Objectives**	4	Provide an explicit statement of the questions and objectives being addressed with reference to their key elements (e.g., population or participants, concepts, and context) or other relevant key elements used to conceptualize the review questions and/or objectives.	1. Introduction, p. 1–2; Abstract, p. 1
**METHODS**			
**Protocol and registration**	5	Indicate whether a review protocol exists; state if and where it can be accessed (e.g., a Web address); and if available, provide registration information, including the registration number.	2.1. Protocol and Registration, p. 2 — registered on OSF Registries (Open-Ended Registration; DOI 10.17605/OSF.IO/2GH63)
**Eligibility criteria**	6	Specify characteristics of the sources of evidence used as eligibility criteria (e.g., years considered, language, and publication status), and provide a rationale.	2.2. Eligibility Criteria, p. 3; Table 1, p. 3
**Information sources**	7	Describe all information sources in the search (e.g., databases with dates of coverage and contact with authors to identify additional sources), as well as the date the most recent search was executed.	2.3. Information Sources and Search Strategy, p. 3
**Search**	8	Present the full electronic search strategy for at least 1 database, including any limits used, such that it could be repeated.	2.3. Information Sources and Search Strategy, p. 3; full verbatim search strings for all four databases in Supplementary Materials (Table S1) and in the OSF registration (DOI 10.17605/OSF.IO/2GH63)
**Selection of sources of evidence**	9	State the process for selecting sources of evidence (i.e., screening and eligibility) included in the scoping review.	2.4. Selection of Sources of Evidence, p. 3
**Data charting process**	10	Describe the methods of charting data from the included sources of evidence (e.g., calibrated forms or forms that have been tested by the team before their use, and whether data charting was done independently or in duplicate) and any processes for obtaining and confirming data from investigators.	2.5. Data Charting and Synthesis, p. 4
**Data items**	11	List and define all variables for which data were sought and any assumptions and simplifications made.	2.5. Data Charting and Synthesis, p. 4
**Critical appraisal of individual sources of evidence**	12	If done, provide a rationale for conducting a critical appraisal of included sources of evidence; describe the methods used and how this information was used in any data synthesis (if appropriate).	*Not applicable — a critical appraisal of individual sources was not conducted, consistent with PRISMA-ScR guidance for scoping reviews (2.1/2.5, p. 2–4)*
**Synthesis of results**	13	Describe the methods of handling and summarizing the data that were charted.	2.5. Data Charting and Synthesis, p. 4
**RESULTS**			
**Selection of sources of evidence**	14	Give numbers of sources of evidence screened, assessed for eligibility, and included in the review, with reasons for exclusions at each stage, ideally using a flow diagram.	3.1. Selection of Sources of Evidence, p. 4; Figure 1, p. 5; Table 2, p. 5
**Characteristics of sources of evidence**	15	For each source of evidence, present characteristics for which data were charted and provide the citations.	3.2. Characteristics of Included Studies, p. 5; Table 3, p. 6

**Critical appraisal within sources of evidence**	16	If done, present data on critical appraisal of included sources of evidence (see item 12).	*Not applicable — critical appraisal was not performed (see item 12)*
**Results of individual sources of evidence**	17	For each included source of evidence, present the relevant data that were charted that relate to the review questions and objectives.	3.3.–3.6., p. 8; Table 4, p. 7
**Synthesis of results**	18	Summarize and/or present the charting results as they relate to the review questions and objectives.	3.3.–3.7., p. 8–9
**DISCUSSION**			
**Summary of evidence**	19	Summarize the main results (including an overview of concepts, themes, and types of evidence available), link to the review questions and objectives, and consider the relevance to key groups.	4. Discussion, p. 10–11
**Limitations**	20	Discuss the limitations of the scoping review process.	4.1. Strengths and Limitations, p. 11
**Conclusions**	21	Provide a general interpretation of the results with respect to the review questions and objectives, as well as potential implications and/or next steps.	5. Conclusions, p. 11
**FUNDING**			
**Funding**	22	Describe sources of funding for the included sources of evidence, as well as sources of funding for the scoping review. Describe the role of the funders of the scoping review.	Funding, p. 12

From: Tricco AC, Lillie E, Zarin W, et al. PRISMA Extension for Scoping Reviews (PRISMA-ScR): Checklist and Explanation. Ann Intern Med. 2018;169:467-473. doi:10.7326/M18-0850.
