# Supplementary material for: Dermoscopy in Monitoring Treatment Response in Scabies: A Scoping Review
Source: J Clin Med. 2026 Sep 12;15(18):7085. doi: 10.3390/jcm15187085 (PMC13607758; doi:10.3390/jcm15187085)
Supplement: Supplementary file 1 [file jcm-15-07085-s001.zip › Table S1.pdf]

Table S1. Search Strategy (as conducted)

Dermoscopy in Monitoring Treatment Response in Scabies: A Scoping Review

Databases searched: PubMed/MEDLINE; Web of Science Core Collection; EBSCO (Academic Search Ultimate); Scopus.
Date all databases were searched: 30 July 2026.
Limits applied: publication years 2016–2026; English language.
Strategy: a deliberate two-concept design (scabies × dermoscopy). No third "monitoring / treatment-response" concept was added as a search filter, to avoid missing studies that perform post-treatment dermoscopic assessment without using that vocabulary in the title, abstract or keywords; the monitoring requirement was applied at the full-text stage.

Concept blocks (search terms, as run)

Concept 1 — scabies: scabies OR "Sarcoptes scabiei"
Concept 2 — dermoscopy: dermoscop* OR dermatoscop* OR videodermoscop* OR videodermatoscop* OR entodermoscop*
Combined: Concept 1 AND Concept 2 (limited to 2016–2026, English).

Full electronic search strategy, per database (as run on 30 July 2026)

Database (platform) — field	Search string / query line (verbatim)	Records retrieved
**PubMed / MEDLINE — Title/Abstract [tiab]**	`(scabies[tiab] OR "Sarcoptes scabiei"[tiab]) AND (dermoscop*[tiab] OR dermatoscop*[tiab] OR videodermoscop*[tiab] OR videodermatoscop*[tiab] OR entodermoscop*[tiab]) AND 2016:2026[dp] AND english[la]`	**143**
**Web of Science Core Collection — Topic (TS)**	`TS=(scabies OR "Sarcoptes scabiei") AND TS=(dermoscop* OR dermatoscop* OR videodermoscop* OR videodermatoscop* OR entodermoscop*) AND PY=(2016-2026) AND LA=(English)`	**173**
**EBSCOhost — Academic Search Ultimate**	`(scabies OR "Sarcoptes scabiei") AND (dermoscop* OR dermatoscop* OR videodermoscop* OR videodermatoscop* OR entodermoscop*) AND LA English`	**195**
**Scopus — TITLE-ABS-KEY**	`TITLE-ABS-KEY(scabies OR "Sarcoptes scabiei") AND TITLE-ABS-KEY(dermoscop* OR dermatoscop* OR videodermoscop* OR videodermatoscop* OR entodermoscop*) AND PUBYEAR > 2015 AND PUBYEAR < 2027 AND LANGUAGE(english)`	**246**
	**Total records identified (all databases)**	**757**

Records after de-duplication and screening

Records identified: 757 (PubMed 143; Web of Science 173; EBSCO 195; Scopus 246)
Duplicates removed: 435
Unique records screened (title/abstract): 322
Excluded at title/abstract: 305
Reports sought for full-text assessment: 17 (1 not retrieved)
Reports assessed for eligibility: 16 (6 excluded, with reasons — see Table 5 of the main text)
Studies included: 10 (backward/forward citation searching added none)

Notes. All four query lines are reproduced verbatim as run on 30 July 2026, in each platform's native syntax. Truncation (*) captures morphological variants (e.g., dermoscop → dermoscopy, dermoscopic; dermatoscop* → dermatoscopy, dermatoscopic). The raw de-duplicated export files (PubMed .txt; Web of Science, EBSCO and Scopus .ris), dated 30 July 2026, are deposited alongside this document and reproduce the record counts stated above.*
