# Supplementary material for: Dermoscopy in Monitoring Treatment Response in Scabies: A Scoping Review
Source: J Clin Med. 2026 Sep 12;15(18):7085. doi: 10.3390/jcm15187085 (PMC13607758; doi:10.3390/jcm15187085)
Supplement: Supplementary file 1 [file jcm-15-07085-s001.zip › Table S2.pdf]

Table S2. Backward- and forward-citation searching (snowballing) supplementing the database search (30 July 2026): studies assessed and verdicts.

Study / source (citation-search direction)	Verdict	Rationale
Seiler et al. 2022 [26] — backward citation (reference list of Ankad et al. [17], their 5th reference)	Excluded	Two pooled vehicle-controlled spinosad RCTs (classic scabies), surfaced from the reference list of Ankad et al. [17]. Excluded: no separable dermoscopic result was reported. Complete cure required both a clinical cure and a microscopic cure; within the microscopic-cure component, absence of mites/eggs/scybala could be demonstrated by microscopy or dermoscopy interchangeably, with a negative dermoscopy result required for burrows. However, no separable dermoscopic appearance, morphology or trajectory is described — dermoscopy is used only as a binary element of a composite cure endpoint, so no dermoscopic finding distinct from the other methods establishes activity or resolution (unlike studies that report such a result even when cure is defined clinically, e.g., Caumes [18]). The operational criterion is therefore not met. Retained in the reference list and cited in the discussion as a large trial that names dermoscopic markers without using them as a monitoring instrument (Gap 2); reference [26].
Haas 2001 [5]; Micali 2004 [6] — backward citation (via the topical reviews [4,7])	Excluded	Primary anchors of dermoscopic monitoring cited by the review literature; published before the 2016–2026 window.
Pietkiewicz et al. 2025, J. Am. Acad. Dermatol. [30] — backward citation (reference of the JAAD viability paper)	Excluded	Methodology/device Technology Pearl on smartphone–dermatoscope integration as a general high-magnification imaging alternative to videodermatoscopy; it does not concern scabies at all (no mention of scabies, mites or treatment), so the population criterion is not met.
Studies citing the 10 included studies and the key UVFD literature — forward citation	Excluded (none new)	Diagnostic sign papers (e.g., "ball sign", "tunnel window sign", mite-gallery unit), case reports with clinical-only follow-up, and efficacy trials without a dermoscopic timepoint. All are captured by the database search and appear in the full screening log; no eligible study was unique to forward citation.
Pietkiewicz et al. 2026, J. Am. Acad. Dermatol. [10] — forward citation (cites Pietkiewicz & Navarrete-Dechent 2023 [28]); also captured by the database search	Excluded	Not unique to citation searching: the same report was also retrieved by the 30 July 2026 database search and excluded at full text (reference [10]; full reason in Table 5). Listed here to document that forward citation surfaced no new eligible study.
Pietkiewicz et al. 2026, Dermatol. Ther. [31] — forward citation (cites Pietkiewicz & Navarrete-Dechent 2023 [28])	Excluded	Narrative review (secondary literature); its scabies section (Figure 13) illustrates that a viable mite is bright green under UVFD whereas a dead mite shows no fluorescence, and states that UVFD can assess mite viability — green mites indicating the need for retreatment and dark ("hyporeflective") mites confirming therapeutic success. This rests on illustrative demonstration and prior work rather than a designed monitoring study — consistent with the "declared rather than designed" finding.
